# Draft Genome Sequence of *Fonsecaea monophora* Strain CBS 269.37, an Agent of Human Chromoblastomycosis

**DOI:** 10.1128/genomeA.00731-16

**Published:** 2016-07-28

**Authors:** Amanda Bombassaro, Sybren de Hoog, Vinicius A. Weiss, Emanuel M. Souza, Aniele C. R. Leão, Flávia F. Costa, Valter Baura, Michele Z. Tadra-Sfeir, Eduardo Balsanelli, Leandro F. Moreno, Roberto T. Raittz, Maria Berenice R. Steffens, Fabio O. Pedrosa, Jiufeng Sun, Liyan Xi, Anamélia L. Bocca, Maria S. Felipe, Marcus Teixeira, Germana D. Santos, Flávio Q. Telles Filho, Conceição M. P. S. Azevedo, Renata R. Gomes, Vânia A. Vicente

**Affiliations:** a Microbiology, Parasitology and Pathology Post-Graduation Program, Department of Pathology, Federal University of Paraná, Curitiba, Paraná, Brazil; b CBS-KNAW Fungal Biodiversity Center, Utrecht, The Netherlands; c Laboratory of Bioinformatics, Professional and Technological Education Sector, Federal University of Paraná, Curitiba, Paraná, Brazil; d Department of Biochemistry, Federal University of Paraná, Curitiba, Paraná, Brazil; e Engineering Bioprocess and Biotechnology Post-Graduation Program, Department of Bioprocess Engineering and Biotechnology, Federal University of Paraná, Curitiba, Paraná, Brazil; f Guangdong Provincial Institute of Public Health, Guangdong Provincial Center for Disease Control and Prevention, Guangdong, China; g Department of Dermatology, Sun Yat-Sen Memorial Hospital, Sun Yat-sen University, Guangzhou, China; h University of Brasilia, Brasilia, Brazil; i Catholic University of Brasilia UCB, Brasilia, Brazil; j Clinical Hospital, Federal University of Paraná, Curitiba, Paraná, Brazil; k Health Science Post-Graduation Program, Department of Medicine, Federal University of Maranhão, Maranhão, Brazil

## Abstract

The black yeast *Fonsecaea monophora* is one of the main etiologic agents of chromoblastomycosis in humans. Its pathogenicity profile is more invasive than that of related *Fonsecaea* species, causing brain infection in addition to (sub)cutaneous infections.

## GENOME ANNOUNCEMENT

The black yeasts are heterogeneous organisms responsible for a wide variety of clinical conditions, ranging from superficial to deep and disseminated infections (1, 2). Chromoblastomycosis is a unique disease characterized by lesions of skin and subcutaneous tissue, leading to a warty appearance with ulcerative, tumorous eruptions. Occasionally, the same fungi manifest systemically in internal organs (3). The disease has three main etiological agents, *Fonsecaea pedrosoi*, *Phialophora verrucosa*, and *Cladophialophora carrionii* (4). Recently, different species have been recognized in *Fonsecaea* as agents of disease (4–7). These agents probably have a life cycle in the environment but have a pathogenic potential. The pathogenicity of *F. monophora* is reflected in low environmental occurrence and high frequency in the human host. Chromoblastomycosis appears to be polyphyletic within a single family of fungi, and is caused by different, rather distantly related species (8). The species *F. monophora* presents a virulence profile that differs from that of other *Fonsecaea* agents of the disease, as it causes cutaneous and subcutaneous chromoblastomycosis but frequently also primary brain infection (9, 10). The epidemiology of the disease has not been fully elucidated; questions related to its infection route, prevalent etiologic agents, and virulence have to be clarified. Total genome sequencing will help to elucidate virulence genes and pathogenicity mechanisms of the agents. Functional analysis of these genes will contribute to finding novel targets for drug development to improve therapy, and will provide further understanding of relevant genes expressed during infection (11).

*Fonsecaea monophora* CBS 269.37, a type strain of the species, was isolated in South America in 1936, redescribed by de Hoog et al. (4), and used in this study. The strain was grown in Sabouraud’s broth, with shaking at 150 rpm at 28°C for 7 days and DNA was extracted by the cetyltrimethylammonium bromide (CTAB) method using phenol-chloroform/isoamyl alcohol. Total DNA was purified with the Microbial DNA ultra-clean kit. Two libraries were constructed using the kit Nextera XT for construction of the paired end library (2 × 300) and library prep kit for Ion Torrent (Thermo, Fisher Scientific) generating over 6 million readings. Genomic sequence reads were generated on the Illumina platform MiSeq (Life Technology) and Ion Torrent PGM platform (Thermo, Fisher Scientific). The reads were assembled *de novo* using SPADES v3.5.0 (12). The draft comprised 324 contigs, with a *N*_50_ of 268,916 bp and the genome size was 35.22 Mb, with a G+C content of 52.22%. Gap closure was performed with FGAP software (13). Protein-coding genes were predicted with GeneMark-ES (14). Annotation for 11,984 predicted genes were assigned based on similarity searches against the nr database using RAFTS3 (15) and InterProScan (16) comparisons. The genome contained 37 tRNAs identified using ARAGORN (17).

The information generated via this genome sequence might provide better understanding of the basic mechanisms of adaptation to its natural habitat, as well as of its pathogenicity and virulence.

### Nucleotide sequence accession numbers.

This whole-genome shotgun project has been deposited in DDBJ/ENA/GenBank under the accession no. LVKK00000000. The version described in this paper is version LVKK01000000.

## References

[1] Queiroz-Telles F, Nucci M, Colombo AL, Tóbon A, Restrepo A 2011 Mycoses of implantation in Latin America: an overview of epidemiology, clinical manifestations, diagnosis and treatment. Med Mycol 49:225–236. doi:10.3109/13693786.2010.539631. 21128710

[2] Seyedmousavi S, Netea MG, Mouton JW, Melchers WJG, Verweij PE, de Hoog GS 2014 Black yeasts and their filamentous relatives: principles of pathogenesis and host defense. Clin Microbiol Rev 27:527–542. doi:10.1128/CMR.00093-13. 24982320 PMC4135901

[3] Vicente VA, Najafzadeh MJ, Sun J, Gomes RR, Robl D, Marques SG, Azevedo CMPS, de Hoog GS 2013 Environmental siblings of black agents of human chromoblastomycosis. Fungal Divers 62:1–17. doi:10.1007/s13225-013-0246-5.

[4] de Hoog GS, Attili-Angelis D, Vicente VA, Van Den Ende AH, Queiroz-Telles F 2004 Molecular ecology and pathogenic potential of *Fonsecaea* species. Med Mycol 42:405–416. doi:10.1080/13693780410001661464. 15552642

[5] Vicente VA, Orélis-Ribeiro R, Najafzadeh MJ, Sun J, Guerra RS, Miesch S, Ostrensky A, Meis JF, Klaassen CH, de Hoog GS, Boeger WA 2012 Black yeast-like fungi associated with lethargic Crab disease (LCD) in the mangrove-land crab, *Ucides cordatus* (*Ocypodidae*). Vet Microbiol 158:109–122. doi:10.1016/j.vetmic.2012.01.031. 22440399

[6] Najafzadeh MJ, Vicente VA, Sun J, Meis JF, de Hoog GS 2011 *Fonsecaea multimorphosa* sp. nov., a new species of *Chaetothyriales* isolated from a feline cerebral abscess. Fungal Biol 115:1066–1076. doi:10.1016/j.funbio.2011.06.007. 21944218

[7] de Azevedo CMPS, Gomes RR, Vicente VA, Santos DWCL, Marques SG, do Nascimento MMF, Andrade CEW, Silva RR, Querioz-Telles F, de Hoog GS 2015 *Fonsecaea pugnacius*, a novel agent of disseminated chromoblastomycosis. J Clin Microbiol 53:2674–2685. doi:10.1128/jcm.00637-15. 26085610 PMC4508443

[8] Vicente VA, Attili-Angelis D, Pie MR, Queiroz-Telles F, Cruz LM, Najafzadeh MJ, de Hoog GS, Zhao J, Pizzirani-Kleiner A 2008 Environmental isolation of black yeast-like fungi involved in human infection. Stud Mycol 61:137–144. doi:10.3114/sim.2008.61.14. 19287536 PMC2610314

[9] Xie Z, Feng P, Zhang J, Li X, Sun J, Lu C, Huang H, Xi L 2012 Molecular cloning, characterization and differential expression of Cdc42 in *Fonsecaea monophora*. Mol Biol Rep 39:839–844. doi:10.1007/s11033-011-0806-2. 21573800

[10] Pindycka-Piaszczyńska M, Krzyściak P, Piaszczyński M, Cieślik S, Januszewski K, Izdebska-Straszak G, Jarząb J, de Hoog GS, Jagielski T 2014 Chromoblastomycosis as an endemic disease in temperate Europe: first confirmed case and review of the literature. Eur J Clin Microbiol Infect Dis 33:391–398. doi:10.1007/s10096-013-1969-7. 24048727

[11] Traeger S, Altegoer F, Freitag M, Gabaldon T, Kempken F, Kumar A, Marcet-Houben M, Pöggeler S, Stajich JE, Nowrousian M 2013 The genome and development-dependent transcriptomes of *Pyronema confluens*: a window into fungal evolution. PLoS Genet 9:e1003820. doi:10.1371/journal.pgen.1003820. 24068976 PMC3778014

[12] Bankevich A, Nurk S, Antipov D, Gurevich AA, Dvorkin M, Kulikov AS, Lesin VM, Nikolenko SI, Pham S, Prjibelski AD, Pyshkin AV, Sirotkin AV, Vyahhi N, Tesler G, Alekseyev MA, Pevzner PA 2012 SPAdes: a new genome assembly algorithm and its applications to single-cell sequencing. J Comput Biol 19:455–477. doi:10.1089/cmb.2012.0021. 22506599 PMC3342519

[13] Piro VC, Faoro H, Weiss VA, Steffens MBR, Pedrosa FO, Souza EM, Raittz RT 2014 FGAP: an automated gap closing tool. BMC Res Notes 7:371. doi:10.1186/1756-0500-7-371. 24938749 PMC4091766

[14] Ter-Hovhannisyan V, Lomsadze A, Chernoff YO, Borodovsky M 2008 Gene prediction in novel fungal genomes using an ab initio algorithm with unsupervised training. Genome Res 18:1979–1990. doi:10.1101/gr.081612.108. 18757608 PMC2593577

[15] Viale RA, Pedrosa FO, Weiss VA, Guizelini D, Tibaes JH, Marchaukoski JN, Souza EM, Raittz RT 31 5 2016 RAFTS3: rapid alignment-free tool for sequences similarity search. bioRxiv. doi:10.1101/055269.

[16] Jones P, Binns D, Chang HY, Fraser M, Li W, McAnulla C, McWilliam H, Maslen J, Mitchell A, Nuka G, Pesseat S, Quinn AF, Sangrador-Vegas A, Scheremetjew M, Yong SY, Lopez R, Hunter S 2014 Inter-ProScan 5: genome-scale protein function classification. Bioinformatics 30:1236–1240. doi:10.1093/bioinformatics/btu031. 24451626 PMC3998142

[17] Laslett D, Canback B 2004 ARAGORN, a program to detect tRNA genes and tmRNA genes in nucleotide sequences. Nucleic Acids Res 32:11–16. doi:10.1093/nar/gkh152. 14704338 PMC373265

